# Adaptation at different points along antibiotic concentration gradients

**DOI:** 10.1098/rsbl.2020.0913

**Published:** 2021-05-12

**Authors:** Mato Lagator, Hildegard Uecker, Paul Neve

**Affiliations:** ^1^ IST Austria, Am Campus 1, 3400 Klosterneuburg, Austria; ^2^ School of Biological Sciences, University of Manchester, Manchester M13 9PT, UK; ^3^ Institute of Integrative Biology, ETH Zurich, 8092 Zurich, Switzerland; ^4^ Research group Stochastic Evolutionary Dynamics, Department of Evolutionary Theory, Max Planck Institute for Evolutionary Biology, 24306 Plön, Germany; ^5^ Biointeractions and Crop Protection Department, Rothamsted Research, Harpenden, Hertfordshire AL5 2JQ, UK; ^6^ Department of Plant and Environmental Sciences, University of Copenhagen, Højbakkegård 9, Tåstrup 2630, Denmark

**Keywords:** antibiotic resistance, drug dose, adaptation

## Abstract

Antibiotic concentrations vary dramatically in the body and the environment. Hence, understanding the dynamics of resistance evolution along antibiotic concentration gradients is critical for predicting and slowing the emergence and spread of resistance. While it has been shown that increasing the concentration of an antibiotic slows resistance evolution, how adaptation to one antibiotic concentration correlates with fitness at other points along the gradient has not received much attention. Here, we selected populations of *Escherichia coli* at several points along a concentration gradient for three different antibiotics, asking how rapidly resistance evolved and whether populations became specialized to the antibiotic concentration they were selected on. Populations selected at higher concentrations evolved resistance more slowly but exhibited equal or higher fitness across the whole gradient. Populations selected at lower concentrations evolved resistance rapidly, but overall fitness in the presence of antibiotics was lower. However, these populations readily adapted to higher concentrations upon subsequent selection. Our results indicate that resistance management strategies must account not only for the rates of resistance evolution but also for the fitness of evolved strains.

## 1. Introduction

The extensive use of antibiotics in medicine and agriculture selects for resistance with detrimental consequences for human health [1]. Furthermore, the rapid evolution of resistance threatens to outpace the discovery of new compounds [2]. To slow the emergence and spread of resistant strains, we need to understand the factors that influence the evolution and maintenance of resistance.

Bacteria may be exposed to widely varying doses of antibiotics. During the course of treatment, antibiotic concentrations are generally high, but vary temporally and spatially across body tissues and organs, exposing bacteria to a variety of selection regimes [3,4]. In the wider environment, bacteria may be exposed to trace concentrations of antibiotics in sewage, rivers and soil [5,6]. Importantly, even these extremely low concentrations of antibiotics can select for resistance mutations [7–9]. Given this heterogeneity in selective pressures, it is important to understand whether (i) different antibiotic concentrations select for different genotypes and (ii) mutations selected at low concentrations can confer resistance to clinically relevant concentrations [10].

Previous studies have demonstrated indirectly that selection at different antibiotic concentrations results in selection for different genotypes [11–15], but direct examinations of how adaptation to one antibiotic concentration correlates with fitness in other environments along an antibiotic concentration gradient have been, with a few exceptions [16,17], generally absent. It is conceivable that the fitness rank of genotypes changes along the antibiotic gradient. For example, it is commonly assumed that adaptation to higher antibiotic concentrations incurs a high fitness cost, meaning that these genotypes are not well adapted at lower concentrations or in the absence of antibiotics [18–20]. However, it is also possible that populations selected at one concentration exhibit the highest fitness across the entire gradient. Which resistant genotype establishes in a bacterial population at a given antibiotic concentration will thus depend on interactions between mutation supply rate and fitness across the concentration gradient. From a resistance management perspective, poor understanding of the relative fitness of resistant strains across the antibiotic dose gradient limits our potential to understand how low concentrations of antibiotics in the environment might select for resistance at clinically relevant doses and prevents evaluation of dose manipulation [21] as a strategy to thwart resistance evolution.

Here, we ask whether: (i) antibiotic concentration impacts the dynamics of resistance evolution, (ii) populations specialize to the concentration they were selected in, (iii) resistance is associated with a fitness cost, and (iv) selection at lower antibiotic concentrations constrains subsequent adaptation to higher concentrations. To address these questions, we evolved six replicate bacterial populations for 28 days (approximately 200 generations) in one of three antibiotic concentrations (the concentration at which growth is half of the growth in the absence of antibiotics—EC_50_; the minimum inhibitory concentration—MIC; and double the MIC—2MIC) (electronic supplementary material, figure S1) and subsequently measured the growth of evolved populations in all three antibiotic concentrations and in the antibiotic-free environment. We employed the same experimental design for three antibiotics (tetracycline, streptomycin and nitrofurantoin) and found that populations selected at higher concentrations exhibit equal or higher fitness across all antibiotic concentrations.

## 2. Material and methods

We only give a brief summary here. Details can be found in electronic supplementary material, S1.

### (a) Evolution experiment

In this study, we exposed *Escherichia coli* K-12 strain to three antibiotics—tetracycline, streptomycin and nitrofurantoin. These antibiotics were chosen because they have a different mode of action and because resistance to them is commonly observed in experimental evolution studies of resistance, as well as clinical and agricultural isolates of *E. coli* [22]. We evolved six replicate populations at three concentrations of each antibiotic (EC_50_, MIC and 2MIC), giving rise to 54 evolving populations, and a ‘wild-type’ population, propagated in the absence of antibiotics. EC_50_ is the half-maximal effective dose of a given antibiotic, i.e. the concentration of an antibiotic that results in half the OD_600_ reached by wild-type cells grown for 24 h in the absence of antibiotics. The MIC is the lowest antibiotic concentration that resulted in no measurable growth after 24 h; 2MIC is double the MIC. We obtained these concentrations by measuring a dose–response of the naive population by transferring 1.5 µl of overnight culture to a serial dilution of each antibiotic, and measuring OD_600_ after 24 h of growth (EC_50_, MIC and 2MIC, respectively, for: tetracycline—0.44, 1.25 and 2.5 µg ml^−1^; streptomycin—14.31, 25 and 50 µg ml^−1^; nitrofurantoin—7.75, 12.5 and 25 µg ml^−1^) (electronic supplementary material, figure S1). During the selection procedure, every 24 h 1.5 µl was transferred to 1.2 ml of fresh medium with the same antibiotic concentration. Since populations evolving at MIC and 2MIC could not grow initially, we immigrated 1.5 µl from the wild-type culture to these populations at every transfer. We immigrated half as much to the populations evolving at EC_50_. After 28 days, we grew all populations in the absence of antibiotics for 24 h, so that all populations reached a similar density. Then, we transferred 1.5 µl of each culture into appropriate media, in order to measure the growth curves of the wild-type and of all evolved populations in (i) the absence of antibiotics and (ii) EC_50_, MIC and 2MIC of the antibiotic in which the populations evolved (three replicate measurements per population, with each set of replicates performed simultaneously), using a BioTek H1 plate reader. From these growth curves, we determined the maximum growth rate, as the greatest slope of the log-transformed growth curve. In addition, we measured the dose–response curves of all evolved populations in the manner described above.

### (b) Statistical analyses

We performed a survival analysis (using the survreg function in R) to determine the effect of the antibiotic concentration on the time to evolve resistance (for this, we called a population ‘resistant’ when after a growth cycle (24 h), it reached an OD_600_ value that was higher by 0.3 than that reached by the wild-type population). We tested whether overall differences in maximum growth rates depended on the selective environment by performing an analysis of variance (anova(lm) function in R) within each tested environment (absence of antibiotics, EC_50_, MIC and 2MIC). To specifically test for differences between every pairwise combination of tested concentration within each antibiotic, we followed up ANOVA with Holm's corrected pairwise *t*-tests. Permutation tests for the maximum growth rates confirmed the conclusions (electronic supplementary material, S2). We performed one-sample *t*-tests (comparing with the wild-type population) in order to determine whether resistance was associated with a cost. For further details, see electronic supplementary material, S1 and S2.

### (c) Further selection at 2MIC

We selected populations that originally evolved at EC_50_ and 2MIC for another nine transfer cycles at 2MIC, using the same protocol as above except without the immigration from the wild-type. We subsequently measured their growth curves at 2MIC and compared the maximum growth rates between them, in the same manner as described above. Furthermore, we tested whether differences in maximum growth rates between populations originally selected at EC_50_ and at 2MIC changed significantly upon further selection. To do so, we randomly paired populations selected at EC_50_ and 2MIC and calculated differences in maximum growth rates for both the original evolved populations and those that had undergone further selection. We performed pairwise *t*-tests for all possible pairings and report average *p*-values.

## 3. Results

### (a) Resistant strains emerge faster at lower concentrations

**Figure 1.**
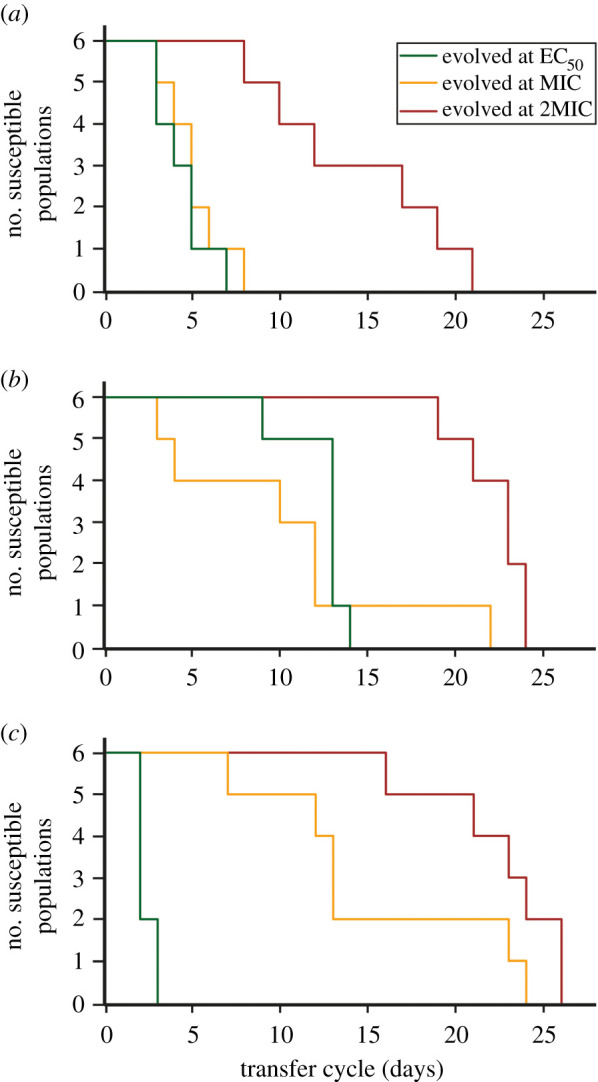
Dynamics of resistance evolution. Kaplan–Meier plots showing the change in the number of susceptible (non-resistant) populations over the course of selection, for populations selected at each of three concentrations (EC_50_—green, MIC—yellow or 2MIC—red) of (*a*) tetracycline, (*b*) streptomycin and (*c*) nitrofurantoin.

We observed the evolution of resistance in all populations by the end of the selection procedure. We found an effect of antibiotic concentration on rates of resistance evolution in all three antibiotic environments (figure 1; for tetracycline: *χ*^2^ = 26.3, *p* < 0.0001; streptomycin: *χ*^2^ = 10.9, *p* < 0.005; nitrofurantoin: *χ*^2^ = 44.3, *p* < 0.0001). In tetracycline and nitrofurantoin, resistance emerged more slowly in populations selected at high concentrations. In streptomycin, resistance evolved most slowly at 2MIC but evolution was faster at MIC than at EC_50_.

### (b) Populations selected at lower concentrations have lower fitness

**Figure 2.**
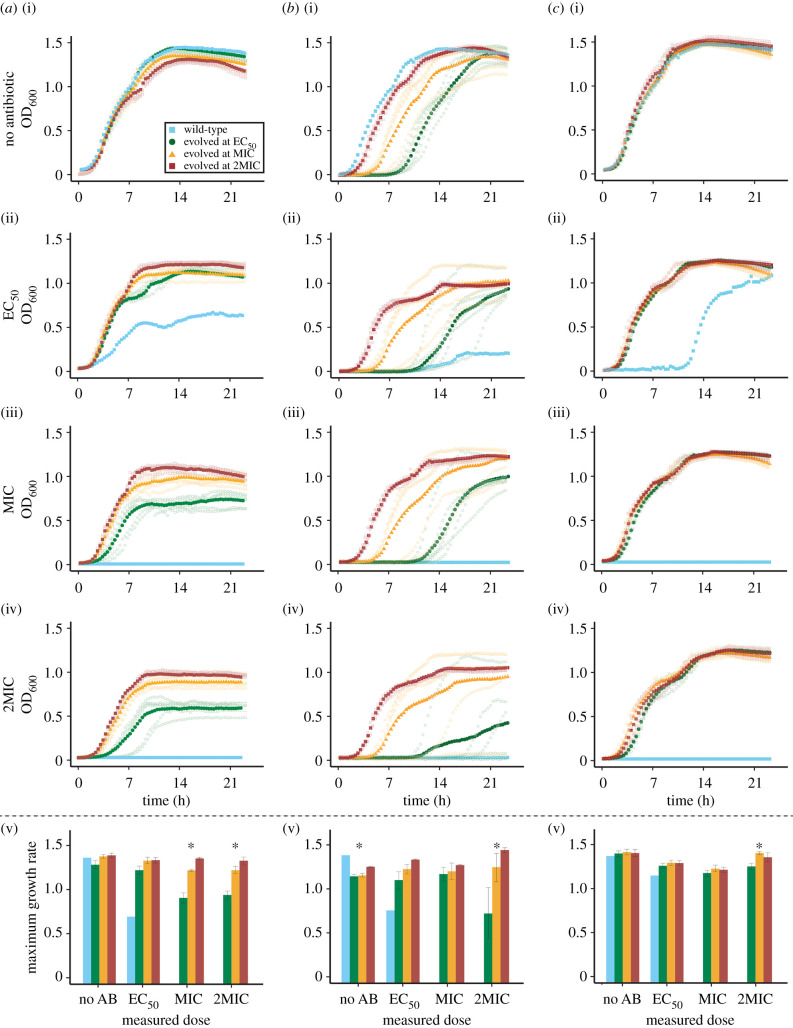
Growth curves and growth rates across antibiotic gradients. Growth curves of evolved populations measured in the absence of antibiotics (no AB), at EC_50_, MIC and 2MIC of the antibiotic; they were selected in (*a*) tetracycline, (*b*) streptomycin or (*c*) nitrofurantoin. Darker dots are the mean OD_600_ across all six populations selected at the same concentration. Each evolved population was measured in three replicates, and the lighter dots show the mean of those three replicates for each evolved population. OD_600_ measurement was taken every 20 min during a 24 h growth cycle. Mean maximum growth rates, as the average over the six populations selected at the same concentration, are shown for each environment. Error bars denote standard errors of the mean, and asterisks mark environments with significant differences between growth rates (based on ANOVA tests). Three replicate measurements of the wild-type population were made. Colours indicate the original concentration that a population was selected in: the wild-type population propagated in the absence of antibiotics (blue), EC_50_ (green), MIC (yellow) and 2MIC (red).

We obtained full growth curves of all evolved populations in EC_50_, MIC and 2MIC of the antibiotic they were selected in, as well as in the absence of antibiotics (figure 2). We estimated fitness as the maximum growth rate in the exponential phase obtained from the growth curves. We also determined three additional features of growth curves—the ‘pseudo lag phase’ duration, the maximum optical density reached and the area under the curve—but avoid drawing conclusions from them due to the difficulty of relating these measures to bacterial fitness (see section ‘Growth Assays’ in electronic supplementary material, S1).

In general, we found that populations selected at higher concentrations had the same or higher fitness across the whole concentration gradient (figure 2; for statistical analyses see electronic supplementary material, S2). Populations selected below MIC still exhibited growth at higher concentrations, albeit of lower overall fitness than the populations selected at higher concentrations. The other three measures of fitness provided further support for this observation (electronic supplementary material, figures S2–S4; S2), which was also evident in the dose–response curves of selected populations (electronic supplementary material, figure S5).

### (c) Fitness costs did not depend on the selective antibiotic concentration

In order to analyse if resistance was associated with a fitness cost, we compared maximum growth rates in the absence of antibiotics between the wild-type population (that had evolved in the absence of antibiotics) and the evolved populations (figure 2). We found no fitness costs in any of the populations selected in tetracycline or nitrofurantoin, while the populations selected in streptomycin exhibited fitness costs (comparing wild-type to populations selected in EC_50_: *t*_5_ = −8.37, *p* < 0.0005; MIC: *t*_5_ = −9.17, *p* < 0.0005; 2MIC: *t*_5_ = −12.00, *p* < 0.0001).

### (d) Further selection at high concentrations leads to assimilation in fitness

Further selection at high concentrations significantly changes fitness differences. Did selection at lower concentrations not only select for lower resistance, but also constrain adaptation to higher antibiotic concentrations? To address this question, we exposed the populations evolved at EC_50_ and 2MIC for nine additional daily transfer cycles (approximately 100 generations) to high concentrations (2MIC) of the same antibiotic they were selected in, and asked if fitness differences reduced. After this further period of evolution, the differences in growth rates between populations originally selected at EC_50_ and at 2MIC had decreased significantly for tetracycline and streptomycin (electronic supplementary material, figure S6; tetracycline: $\bar{p}\approx 0.008$, streptomycin: $\bar{p}\approx 0.026$). A caveat of this analysis is that the growth rates of the original populations and those that had undergone further selection were measured on different days, and growth rates were generally larger for the former than for the latter. Performing the analysis on growth rates scaled with the mean growth rate of populations selected at 2MIC led to similar results (tetracycline: $\bar{p}\approx 0.020$, streptomycin: $\bar{p}\approx 0.025$, nitrofurantoin: $\bar{p}\approx 0.061$). For all antibiotics, differences in growth rates were insignificant following the period of additional selection (although differences remain significant for tetracycline, if the size of the sliding window is reduced from 7 to 6 points: *t*_5_ = −1.92, $\bar{p}\approx 0.042$). Similarly, the differences in other measures of fitness changed (electronic supplementary material, figures S7–S9; S2.5). Interestingly, two populations that had originally been selected at EC_50_ in streptomycin and that were not able to measurably grow at 2MIC were able to adapt during this phase of selection despite their poor starting performance. These results indicate that selection by sub-lethal antibiotic concentrations poses an inherent risk, as rapidly emerging resistance can be difficult to control by subsequent increases of antibiotic concentrations.

## 4. Discussion

Bacterial populations selected at higher antibiotic concentrations were at least as fit or fitter across the entire gradient compared with populations that were exposed to lower antibiotic concentrations (figure 2). This means that, while resistance emerges more slowly at higher antibiotic concentrations (figure 1) [23,24], the populations selected under those conditions typically exhibited higher fitness. While on average less fit across the whole gradient, the populations selected below MIC were able to grow at and above MIC, and rapidly became similar in fitness to populations selected at high concentrations when further selected at higher concentrations.

These observations raise a question—why were the genotypes (mutations) that were selected at higher concentrations not apparently also selected at lower concentrations? Such evolutionary dynamics could result from the distribution of mutational effects (DME). Many experimental estimates of DMEs show that small-effect mutations are more abundant than mutations of large effect [25–29]. It seems plausible that mutations that provide resistance to high levels of antibiotics appear at a lower frequency than the mutations that provide a sufficient benefit only to survive low antibiotic concentrations [17,30]. Thus, at low concentrations, ‘low-resistance’ mutations may arise and become abundant before a rare ‘high-resistance’ mutation is even generated. Once low-resistance mutations have reached high frequency, the fixation of high-resistance mutations is less likely since it needs to compete with a well-adapted genotype. At higher concentrations, those low-resistance mutations confer a smaller fitness benefit, resulting in them being less frequent or absent. Under such conditions, when the competition from low-resistance mutations is reduced, high-resistance mutations that are less frequent can take over the population.

Two recent studies by Harmand *et al.* explored the question of how antibiotic concentration influences resistance evolution, albeit by adopting different approaches [16,17]. In the first study, Harmand *et al* [17] characterized resistance mutations obtained through fluctuation assays along an antibiotic gradient, finding that resistance mutations screened at high concentrations have the highest fitness across all concentrations. In the second study, they used these already resistant strains to further select for resistance along an antibiotic gradient [16]. There are several critical differences between these and our study: (i) Harmand *et al*. selected for resistance only above MIC, while we also selected below MIC, allowing for de novo evolution of resistance; (ii) our study falls in between the two studies since it encompasses the entire evolutionary process (appearance of first mutations and subsequent adaptation). The set-up considered in our study corresponds to adaptation to a sink environment with immigration from an antibiotic-free reservoir as may happen during antibiotic treatment with imperfect drug penetration. In contrast with Harmand *et al*. [16], who identified that continued exposure to higher concentrations of antibiotics led to dose specialization, we found that generalists emerged readily at higher concentrations and had higher fitness across the entire gradient (figure 2). Together, these studies indicate that the effect of the first mutation step is dominant (at least at the time scales considered in our study) and point to the complex interplay between antibiotic concentration and evolutionary outcomes.

While resistance to many antibiotics in *E. coli* emerges through point and other chromosomal mutations [31], this is not the only means of acquiring resistance. Resistance is often inherited through horizontal gene transfer, as commonly observed for tetracycline resistance [32]. Furthermore, resistance can arise through a range of different mechanisms, such as modification of the drug target or drug metabolism [33]. Finally, which mechanisms fix in the population also depends on the specific strain under selection [34]. All these factors can alter the course of resistance evolution by, for example, imposing different costs and using different pathways to compensate for those costs [35,36]. In this study, we did not characterize the mechanisms of resistance that underpin the reported adaptations, and we only considered evolution through chromosomal mutations in one strain of *E. coli*. More complete understanding of how antibiotic concentration impacts resistance evolution must further expand on our work to account for the similarities and differences between various mechanisms of resistance and means of their inheritance.

Manipulations of antibiotic concentrations, as well as pesticides and other xenobiotics, form an important resistance management strategy [21,37–41]. So far, the primary focus has been on rates of resistance evolution, where the traditional approach is to use high concentrations [42,43], although this view has been challenged more recently [39,41,44]. Considering the fitness of the evolved strains adds another dimension to the problem of optimal antibiotic dosing. We show that selection at high concentrations results in a dilemma: resistant populations with higher fitness emerge more slowly but display—at least at the time scales considered here—higher levels of resistance. Careful consideration of both rates of resistance emergence and fitness of resistant strains is necessary when adopting concentration manipulation as a management strategy.

## Data accessibility

The data presented in this work can be found at https://doi.org/10.5061/dryad.ghx3ffbnd [45]. For the scripts used to analyse the data, please contact the authors. The data are also provided in the electronic supplementary material.
